# Mesenchymal cell migration on one-dimensional micropatterns

**DOI:** 10.3389/fcell.2024.1352279

**Published:** 2024-04-16

**Authors:** Johannes C. J. Heyn, Joachim O. Rädler, Martin Falcke

**Affiliations:** ^1^ Fakultät für Physik, Ludwig-Maximilians-Universität München (LMU), Munich, Germany; ^2^ Max Delbrück Center for Molecular Medicine in the Helmholtz Association, Berlin, Germany; ^3^ Department of Physics, Humboldt University, Berlin, Germany

**Keywords:** cell migration, biophysics, single cell dynamics, 1D, one-dimension, confinement, micropattern, mesenchymal

## Abstract

Quantitative studies of mesenchymal cell motion are important to elucidate cytoskeleton function and mechanisms of cell migration. To this end, confinement of cell motion to one dimension (1D) significantly simplifies the problem of cell shape in experimental and theoretical investigations. Here we review 1D migration assays employing micro-fabricated lanes and reflect on the advantages of such platforms. Data are analyzed using biophysical models of cell migration that reproduce the rich scenario of morphodynamic behavior found in 1D. We describe basic model assumptions and model behavior. It appears that mechanical models explain the occurrence of universal relations conserved across different cell lines such as the adhesion-velocity relation and the universal correlation between speed and persistence (UCSP). We highlight the unique opportunity of reproducible and standardized 1D assays to validate theory based on statistical measures from large data of trajectories and discuss the potential of experimental settings embedding controlled perturbations to probe response in migratory behavior.

## 1 Introduction

The motion of eukaryotic cells is essential for embryonic development, wound healing, immune response, and tumor metastasis (Pollard and Borisy, 2003). Much effort has been devoted to the study of mesenchymal migration with prototypical *in vitro* motion of cells on plane adhesive substrates. Mesenchymal cell migration starts with polarization breaking the spatial symmetry and the formation of protrusion of a thin sheet of cytoplasm (0.1–0.3 µm thick) covering tens to hundreds of square micrometers (Abercrombie et al., 1971; Abercrombie, 1980; Ridley et al., 2003; Dawes and Edelstein-Keshet, 2007; Yam et al., 2007; Insall and Machesky, 2009; Lomakin et al., 2015). It is mechanically stabilized by adhesion with the substrate (Huttenlocher et al., 1996; Lauffenburger and Horwitz, 1996; Palecek et al., 1997; Sheetz et al., 1998; Vicente-Manzanares et al., 2009; Parsons et al., 2010; Ross et al., 2013; Burridge and Guilluy, 2016; Li et al., 2023). The lamellipodium behind its protruding contour is constructed from a network of actin filaments (Small and Celis, 1978; Small et al., 1978; Svitkina et al., 1997; Small et al., 2002; Pollard, 2003; Oosterheert et al., 2022; Reynolds et al., 2022; Bibeau et al., 2023; Carman et al., 2023). Polymerization of filament barbed ends at the leading edge of the lamellipodium generates motion and pushes the edge forward (Wang, 1985; Prass et al., 2006; Ananthakrishnan and Ehrlicher, 2007; Heinemann et al., 2011; Ridley, 2011; Abu Shah and Keren, 2013; Bisi et al., 2013; Kage et al., 2017). Further back, the pointed ends depolymerize and replenish the pool of actin monomers (Small et al., 2002; Pollard, 2003). Once cells are moving, their shape is determined by internal force generation patterns and adhesion (Schaub et al., 2007; Keren et al., 2008; Bretschneider et al., 2009; Barnhart et al., 2011; Shao et al., 2012; Cramer, 2013; Ziebert and Aranson, 2013).

Quantitative analysis of cell motion reveals systematic relations between characteristics of cell motion and parameters affecting motion. Relations describing the response of a system to external parameters or relations among parameters are called constitutive relations in Physics and Engineering. Many cell types show both the adhesion-velocity relation and the universal correlation between speed and persistence (UCSP). The dependency of the cell velocity on adhesion exhibits a velocity maximum at intermediate strength, and slower velocities both at weak and strong adhesion (Palecek et al., 1997). This has been confirmed for many cell types (CHO cells (Palecek et al., 1997), PtK1 cells (Gupton and Waterman-Storer, 2006), keratocytes (Barnhart et al., 2011), myoblasts (DiMilla et al., 1991), smooth muscle cells (DiMilla et al., 1993), glioma cells (Klank et al., 2017), HuH-7 cells (Hu et al., 2022), MDA-MB-231 cells (Schreiber et al., 2021). Results on the UCSP, describing the relation between cell velocity and persistence time, suggest it to be of similar universality (Maiuri et al., 2015; Jerison and Quake, 2020; Amiri et al., 2023; Leineweber and Fraley, 2023). The faster cells move the more persistent they move. Maiuri et al. report this observation for many different cell types and suggest persistence time to depend exponentially on cell velocity (Maiuri et al., 2015). Leineweber and Fraley report that most of the MDA-MB-231 and HT1080 cells in their study obey the coupling of speed and persistence, but some cells deviate from it (Leineweber and Fraley, 2023). The study looks at motion in 3D extracellular matrix (ECM). Cells deviating from the speed-persistence coupling showed a loss of adhesion (Leineweber and Fraley, 2023). If the pore size of the ECM is smaller than 10% of the nucleus cross section, cell motion relies on matrix degradation (Wolf et al., 2013). Leineweber and Fraley suggest that the lack of speed-persistence coupling is also due to loss of the coordination between matrix degradation and protrusion in these cells. Hence, this study suggests that the dysfunction of modules required for motility entails loss of speed-persistence coupling but fully functional cells obey it.

Another general observation is that both the shape and the motile state of cells is highly dynamic. Cells stop and start to move again, develop new protrusions, change direction (Giannone et al., 2004; Döbereiner et al., 2006; Enculescu et al., 2008; Gholami et al., 2008; Koestler et al., 2008; Bretschneider et al., 2009; Enculescu et al., 2010; Burnette et al., 2011; Doubrovinski and Kruse, 2011; Allard and Mogilner, 2013; Ziebert and Aranson, 2013; Gerhardt et al., 2014; Zimmermann and Falcke, 2014; Barnhart et al., 2017; Beta and Kruse, 2017; Park et al., 2017; Bolado-Carrancio et al., 2020; Hennig et al., 2020; Lee et al., 2022). In addition to these states of motion, states distinguished by the dynamic regime of front protrusion and cell back and/or back protrusion exist. Stationary and oscillatory dynamic regimes with one or several protrusions have been observed, and caused a surge of interest in multistability in cell motility (Verkhovsky et al., 1999; Kozlov and Mogilner, 2007; Hawkins et al., 2011; Tjhung et al., 2012; Ziebert et al., 2012; Lomakin et al., 2015; Ruprecht et al., 2015; Holmes et al., 2017; Park et al., 2017; Bolado-Carrancio et al., 2020; Hennig et al., 2020; Ron et al., 2020; Sens, 2020; Amiri et al., 2023).

Multistability of dynamic states with its state transition dynamics, the biphasic adhesion-velocity relation and the UCSP appear to describe the motile behavior of many different cell types (DiMilla et al., 1993; Palecek et al., 1997; Gupton and Waterman-Storer, 2006; Kozlov and Mogilner, 2007; Tjhung et al., 2012; Ziebert et al., 2012; Maiuri et al., 2015; Holmes et al., 2017; Bolado-Carrancio et al., 2020; Ron et al., 2020; Sens, 2020; Schreiber et al., 2021). While they have been discovered in studies of migration in 1, 2 or 3D, we feel that 1D studies made it very obvious that they are independent observations, i.e., cells in all moving dynamic states obey the adhesion-velocity relation and UCSP, and protrusion oscillations occur independently from direction changes. The generality and concurrency of multistability and the constitutive relations strongly suggest that a single mechanism can explain all of them.

The confinement imposed by one-dimensional (1D) micropatterns restricts protrusions to the two ends of a cell. This simplifies the analysis of the observations described above. With this review we would like to illustrate that 1D migration allows for easier identification of dynamic phenomena and for collecting large amounts of trajectories which provide the basis for formulating stringent biophysical models that explain the emergence of migratory states and universal behavior.

## 2 The use of one-dimensional microlanes for cell motility analysis

**FIGURE 1:**
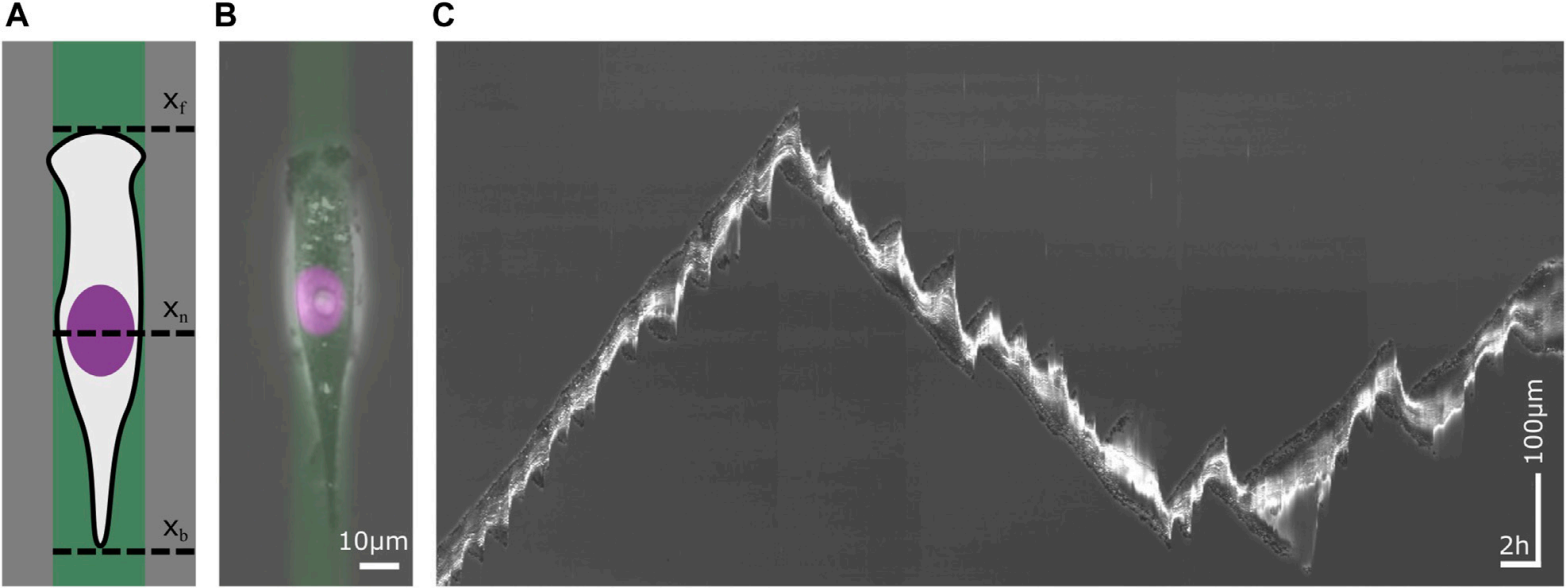
Single cell migration on a 1D micropattern. 1D micropatterns facilitate the study of mesenchymal cell migration by enabling the acquisition of large statistics. **(A)** Schematic sketch of a cell on a lane that has been functionalized with an extracellular matrix (ECM) protein. The migration of the cell is defined by the position of its front x_f_, its nucleus x_n_ and its back x_b_ over time. **(B)** A human breast cancer cell (MDA-MB-231) on a fibronectin (FN) lane. The phase contrast image visualizes the contour of the cell. The nucleus has been stained violet and the ECM protein green. Scale bar 10 µm. **(C)** Kymograph of a migrating cell whose trajectory displays changes in velocity and direction as well as in cell length. Time runs from left to right. The vertical axis represents the position along the center of a micropatterned lane. Horizontal scale bar 1h, vertical scale bar 100 µm.

Single cell migration on 1D micropatterns is considered as a model for cell motion on fibers in the 3D extracellular matrix in tissues (Cukierman et al., 2001; Doyle et al., 2009; Fraley et al., 2012; Doyle et al., 2013; Yamada et al., 2022). It also presents an advantageous approach for streamlining migratory behavior and enabling high-throughput analysis (Maiuri et al., 2012; Lautenschläger and Piel, 2013; Ruprecht et al., 2017). In particular, motion of mesenchymal migration is restricted by an adhesive pattern, as shown in Figure 1A. Adhesive patterns are typically functionalized with an extracellular matrix (ECM) protein such as fibronectin or collagen while the space in between patterns is blocked using cell-repellent block-copolymers such as poly(L-lysine) grafted poly(ethylene glycol) (PLL PEG) (Schreiber et al., 2016; Schuster et al., 2016; Ljepoja et al., 2019; Zhou et al., 2020; Monzo et al., 2021; Kim et al., 2023). The width of the 1D microlanes is a critical parameter and determines the mode of migration (Schreiber et al., 2016; Ljepoja et al., 2019). Typically the lane width is chosen to be of the order of magnitude of the size of the cell nucleus, in which case cells move in integrin dependent mesenchymal migration mode. Live-cell time-lapse imaging is the primary method to acquire data in cell migration studies. An example of a phase contrast image superimposed with fluorescence is shown in Figure 1B. In this example, the micropattern is fluorescently labeled such that the geometry of the confinement is captured. Also, the position of the cell nucleus is tractable using fluorescent labeling. Wide field images provide information about the shape of the cell. Fluorescent cytoskeleton markers are used to determine intracellular structures and activity such as the actin cortex or microtubule.

The acquisition of large data sets of cell trajectories is possible since micropatterns provide standardized and reproducible boundary conditions for cell motion, which facilitate automated image analysis. In scanning time-lapse mode, numerous adjacent view fields are sequentially imaged, with each view field site visited within a single interval of the time-lapse mode, enabling the acquisition of hundreds of migrating cells. In 1D confinement, analysis of migratory cell dynamics is reduced to trajectories of distinctive points over time x_n_(t), such as the trajectory of the cell nucleus. For a minimalistic description of cell shape dynamics, the positions of front and back of the cell, x_f_(t) and x_b_(t), are sufficient to follow cell length over time. Long-term imaging using incubation stages makes it possible to follow the migratory dynamics of migrating cells over a long period of time, see kymograph in Figure 1C. The time frame of a single cell trajectory is usually constrained by the period of cell division. As a slice through a time stack along the time-axis, kymographs visualize the movement of cells in a single picture.

The standardized conditions and large statistics facilitate the comparison of migratory behaviors across different cell lines (Maiuri et al., 2012). Not only does the mean cell speed and mean persistence time vary between cell lines but also the fraction of time cells spent in a motile state and the rate of transitions between these states (Maiuri et al., 2012; Amiri et al., 2023). The quantification of state transitions that are rare is only possible with large ensembles of single cell trajectories. Typically, in a single experiment over 2 days about 1,000 cells yield about 20.000 h of total trajectory length. The comparison of many cell lines makes it possible to find constitutive relations of cell motility such as the universal coupling of cell speed and cell persistence (Maiuri et al., 2015; Schreiber et al., 2021; Amiri et al., 2023).

## 3 Microfabrication of 1D migration platforms

Micropatterns confining cell migration require the fabrication of substrates with defined areas that are functionalized by cell adhesive proteins (ligands) and that are surrounded by a passivation which blocks cell attachment. Micropatterning techniques enable controlled experimental conditions, including pattern geometry, ligand density and substrate stiffness. Various geometries for confined migration have been reported, including lanes of varying width, short stripes, rings and zig-zag patterns, with microlanes emerging as a *de facto* standard for migratory assays (O’Neill et al., 1990; Matsuda and Sugawara, 1996; Levina et al., 2001; Pouthas et al., 2008; Maiuri et al., 2012; Schreiber et al., 2016; Mohammed et al., 2019). Two fabrication techniques have proven particularly useful in recent years: microcontact printing and photopatterning (Kramer et al., 2013; Piel and Théry, 2014a; Piel and Théry, 2014b; Ruprecht et al., 2017).

### 3.1 Microcontact printing

**FIGURE 2:**
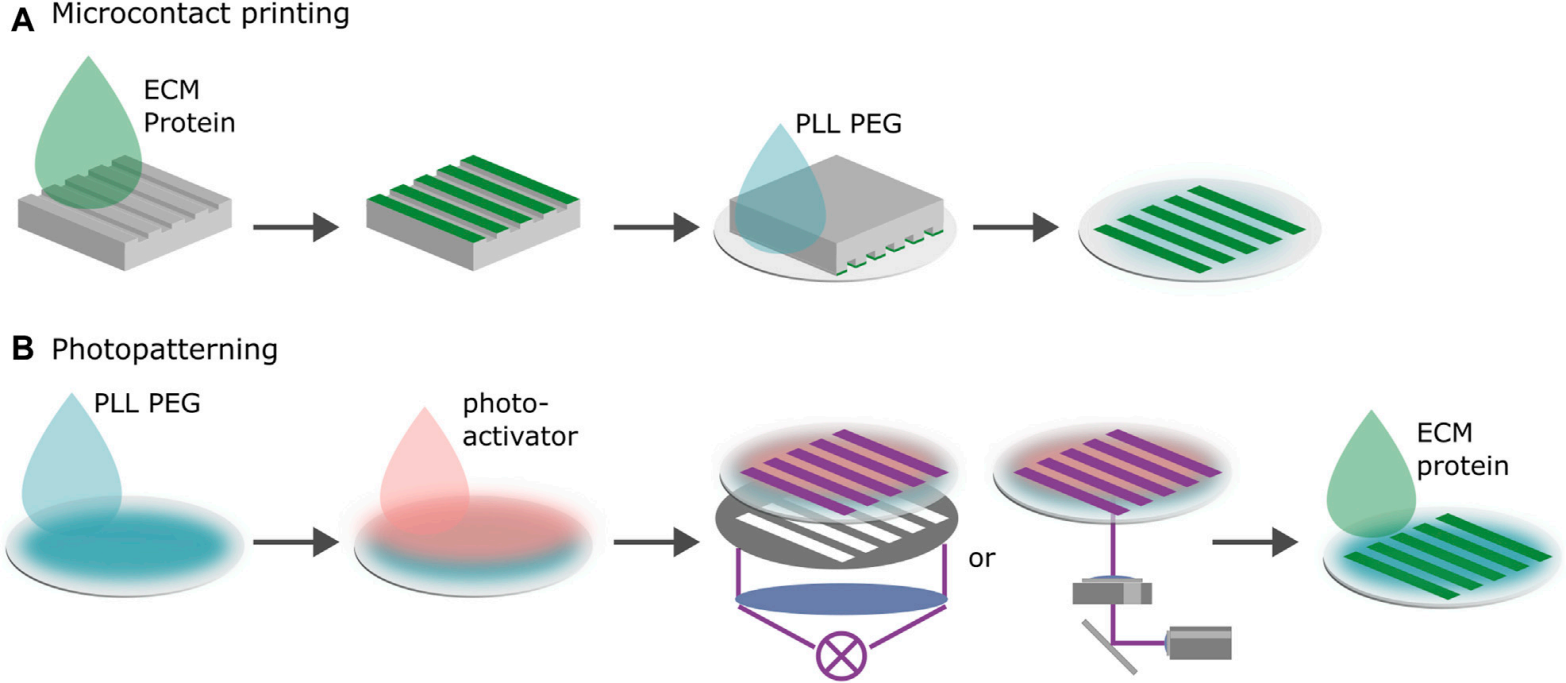
Microfabrication of 1D migration platforms. **(A)** Microcontact printing. Proteins are transferred to the negatively charged substrate via a PDMS stamp in the shape of the desired pattern. Negative space is backfilled with poly(L-lysine) grafted poly(ethylene glycol) (PLL PEG). The surface chemistry of the stamp and the substrate determine the transfer efficiency. For the proteins to transfer onto the substrate, binding to the new surface must be more energetically favorable than staying on the stamp. **(B)** Photopatterning. First, the substrate is passivated with proteins that cells do not adhere to. Second, a photoactive layer is added. Third, the desired pattern is either illuminated through a photomask or by scanning the substrate using spatially modulated UV-light. The photoactive layer removes the surface passivation upon treatment by UV-illumination rendering the substrate locally susceptible to ECM proteins. Last, the protein is added to the substrate and adheres to the treated areas.

Microcontact printing transfers proteins to the substrate via a stamp in the shape of the desired micropattern, see Figure 2A. Stamps are typically produced by pouring a polymer such as PDMS in a negative mold consisting of a Si wafer coated with photoresist. The polymer is cured, cut into stamps, the stamps are incubated with proteins and then placed with the protein coated side on the substrate to transfer the protein. In most cases the protein is simply physisorbed on the surface. The space in between the adhesive patterns can be blocked or passivated by backfilling the negative space of the stamp with a blocking solution. The most common non-fouling system that is used to block protein adsorption is poly(ethylene glycol) (PEG) (Falconnet et al., 2006). Microcontact printing works for various ligands and on a variety of substrates, such as gold, silver, metal-oxide surfaces, glass and various plastic substrates. This makes it possible to use similar protocols with only slight adaptations to test the effect of different substrates and different substrate stiffnesses on cell migration. Once established, microcontact printing provides a reliable, economical method to produce micropatterns (Tan et al., 2004; Piel and Théry, 2014b; Piel and Théry, 2014a; D’Arcangelo and McGuigan, 2015; Vercurysse et al., 2022).

### 3.2 Photopatterning

Photolithographic patterning techniques usually require the substrate to be treated with a blocking or passivation solution and a photo-activator, see Figure 2B (Bélisle et al., 2008; Bélisle et al., 2009; Azioune et al., 2009; Doyle et al., 2009; D’Arcangelo and McGuigan, 2015; Ricoult et al., 2015). Upon exposure of the photoactive layer to ultraviolet light (UV) or near UV light, the passivation layer is removed by a photoscission mechanism rendering the substrate locally susceptible to ligands. One can achieve the same effect without a photo-activator by deploying deep UV (Azioune et al., 2010). Illuminating an area in the shape of the pattern using either a mask or scanning it with a UV beam results in the removal of the passivation layer in a negative pattern. This pattern is then filled with the desired ligands which adhere to the substrate. Maskless projection lithography deploys digital micromirror devices (DMD) to spatially modulate the light. While UV illumination via masks facilitates the scaling of the production of highly reproducible patterns, maskless methods shine when it comes to rapid prototyping. Pattern geometries can easily be tested by simply changing the digital design of the pattern without the need to produce a new photo-mask (Strale et al., 2016). The modulation of ligand density which is commonly used to tune the strength of cell adhesion is easier with photopatterning than with microcontact printing protocols because the density depends on the illumination dose which is readily controlled. However, photopatterning can only be deployed on thin, UV-transparent substrates which renders patterning gel coated substrates challenging.

## 4 Basic observations with cells in 1D confinement

1D assays have been used to characterize a variety of parameters of cell motility like velocity, traction force and response to gradients (Doyle et al., 2013). We summarize basic observations on 1D substrates in this section. Cell behavior is affected by the dimensionality of the substrate (Doyle et al., 2013; Yamada et al., 2022). Cells migrating on ECM fibers or thin fibronectin lanes have a uniaxial shape (Weiss and Garber, 1952; Doyle et al., 2009; Schuster et al., 2016). They lose the ability to orient along fibrillar structures in the absence of microtubules (Doyle et al., 2009). They move on fibers in 3D about 1.5times faster than in 2D (Cukierman et al., 2001) and even more than 1.5times faster on 1.5 µm wide fibronectin lanes (Doyle et al., 2009). The effect is even stronger with human keratinocytes (Doyle et al., 2009). The velocity of fibroblasts showed a biphasic response to line width with maximal velocity at 2.5 µm and smaller velocities on thinner and broader lanes (Doyle et al., 2009). Human umbilical vein endothelial cells (HUVECs) and fish epithelial keratocytes showed increasing velocity with increasing lane width (Schuster et al., 2016; Mohammed et al., 2019).

The cell velocity may be correlated with other cell properties. A study by Leal-Egaña et al. revealed a correlation between cell length, cell speed and the traction energy spent while deforming an elastic substrate with MCF10A human breast cells (Leal-Egaña et al., 2017). Short cells are rather fast and long cells rather slow (Leal-Egaña et al., 2017; Hennig et al., 2020). Short cells spend less energy in deforming the substrate than long cells (Leal-Egaña et al., 2017). This correlation may indicate that more contractile units are active in long than in short cells. Tumorigenic MCF10A cell lines (with constitutively active ErbB2 and knockdown of the β subunit of casein kinase 2) and cells with TGF β applied exhibited each specific distributions of cell length, speed and traction energy, however the correlations between cell length, speed and traction energy were approximately conserved (Leal-Egaña et al., 2017).

The motion of cells responds to environmental cues. Several studies suggest that cells prefer to move into the area where they can adhere the tightest. In our own study we showed that MDA-MB-231 cells facing the boundary of two regions with differential fibronectin density move preferentially (but not exclusively) into the region with higher density (Schreiber et al., 2021; Amiri et al., 2023). We explained this behavior by a biophysical model suggesting an increase of the friction between retrograde flow and adhesion structures due to increased fibronectin density (Schreiber et al., 2021; Amiri et al., 2023). Similarly, d’Alessandro et al. report that MDCK epithelial cells prefer to stay in areas with high fibronectin density (d’Alessandro et al., 2021). Fibroblasts facing the boundary between a 2D region and a 1D lane prefer to stay in the 2D region because they can exert larger traction force there (Chang et al., 2013). Even if they need to traverse a gap where they cannot adhere, they explore the other side by filopodia and prefer to move into the direction where they can stably adhere most filopodia (Caballero et al., 2014; Schreiber et al., 2016). Those three different “implementations” of the preference for tight adhesion suggest it to be a basic property.

Adhesion affects the cell state via integrin signaling and causes structures inside cells which are stationary in the lab frame of motion. The friction between F-actin retrograde flow and these structures obeys the clutch mechanism. It is a basic observation in mesenchymal motility and has been confirmed in a variety of experimental studies (Mitchison and Kirschner, 1988; Suter and Forscher, 2000; Jurado et al., 2005; Hu et al., 2007; Chan and Odde, 2008; Gardel et al., 2008; Gardel et al., 2010; Aratyn-Schaus and Gardel, 2010; Li et al., 2010; Craig et al., 2015). Friction increases with retrograde velocity up to a critical value. The clutch between the F-actin network and structures stationary in the lab frame of reference engages in that velocity range. The clutch disengages at a critical velocity and the friction force drops. The picture explaining this disengagement is that bonds causing friction force break faster than they form (Schallamach, 1966; Grosch and Bowden, 1997). This stick-slip-type behavior is a versatile phenomenon known with many different physical systems. It generates sound in stringed bowed instruments (Ebeling, 1989; Popp and Stelter, 1990), causes earthquakes (Brace and Byerlee, 1966) and wear in materials (Schallamach, 1966) and articular joints (Lee et al., 2013), and robustly generates oscillations (Ebeling, 1989; Filippov et al., 2004). Whether this oscillation mechanism is compatible with the oscillatory characteristics of cell motility is an interesting question for modeling.

The clutch mechanism may apply to protrusions at both ends in many cells on 1D structures (Guetta-Terrier et al., 2015; Monzo et al., 2016; Hennig et al., 2020; Ron et al., 2020; Amiri et al., 2023). The existence of protrusions at both ends poses the question for differences between them and the interaction between both ends, which has been addressed in several studies. Cells migrating in 1D exert traction forces at the front and rear (Han et al., 2016; Hennig et al., 2020). The magnitude of forces at front and rear is very similar (Han et al., 2016; Hennig et al., 2020). It exceeds the force required to move cells by orders of magnitude (Ridley et al., 2003). Changes in traction forces at one end are not correlated to force changes at the other one, suggesting that contraction at the front may not be the main driver for rear retraction (Han et al., 2016). Similarly, changes of traction forces of fibroblasts at one end upon onset of motion were not balanced by changes of the traction forces at the other end despite them being much larger than the force required to move the cell (Hennig et al., 2020). That entailed substantial cellular force asymmetry in terms of traction forces (Hennig et al., 2020).

The forces acting at protrusion edge membranes are in the order of magnitude of 0.1 nN/(µm edge contour length) and are much smaller than traction forces (Prass et al., 2006; Heinemann et al., 2011; Zimmermann et al., 2012) and closer to the force required to move the cell (Ridley et al., 2003). MDA-MB-231 and other cells on 1D lanes have protrusions at both ends most of the time (Hennig et al., 2020; Amiri et al., 2023) allowing for studying directly how forces exerted by protrusions affect other protrusions. Rearward protrusions affect front motion only very little (Hennig et al., 2020; Amiri et al., 2023). Similarly, Doyle et al. report little reduction of protrusion due to inefficient tail retraction (Doyle et al., 2009). Long lasting rearward protrusions at the back increase the propensity of a direction reversal (Amiri et al., 2023). Schreiber et al. described front rear interaction by an elastic spring, as many modeling studies do. Such a force might be caused by volume conservation in 1D motion. It reproduces well the length adaptation of MDA-MB-213 cells on different fibronectin densities and when crossing density steps when an elastic constant of about 0.001 nN/μm per μm cell width (i.e., 0.001 nN/μm^2^) was used (Schreiber et al., 2021). These results from several groups suggest rather weak front-back interaction in the sense that not each protrusion immediately affects all the other ones on the time scale of transmission of elastic forces, but front-back interaction is still sufficient to affect propensities for protrusion collapse if protrusions compete.

This leads to the question of the function of contractile structures often seen aligned in the direction of motion. Han et al. suggest that “contractile forces support the assembly of stress fibers and adhesions via stabilizing molecular bonds in them” (Han et al., 2016). Adhesion regulation happens at the leading and at the trailing edge but less in between (Doyle et al., 2009). Doyle et al. found that adhesion structures of fibroblasts in 1D migration last longer than those of cells on 2D substrate and Blebbistatin induces loss of 1D adhesion stability (Doyle et al., 2012). An analysis of the response of the adhesion-velocity relation of MDA-MB-231 cells to Blebbistatin suggested also the conclusion that contraction affects migration rather as a feedback mechanism in adhesion formation than by direct action on the retrograde flow (Schreiber et al., 2021). All of these results support the idea of a role of myosin contraction mainly in force dependent stabilization of adhesions. Doyle et al. suggest that force dependent stabilization of adhesions is an important factor for promoting migration in restricted environments, since adhesion cannot be strengthened by an increase of adhesive area (Doyle et al., 2012).

**FIGURE 3:**
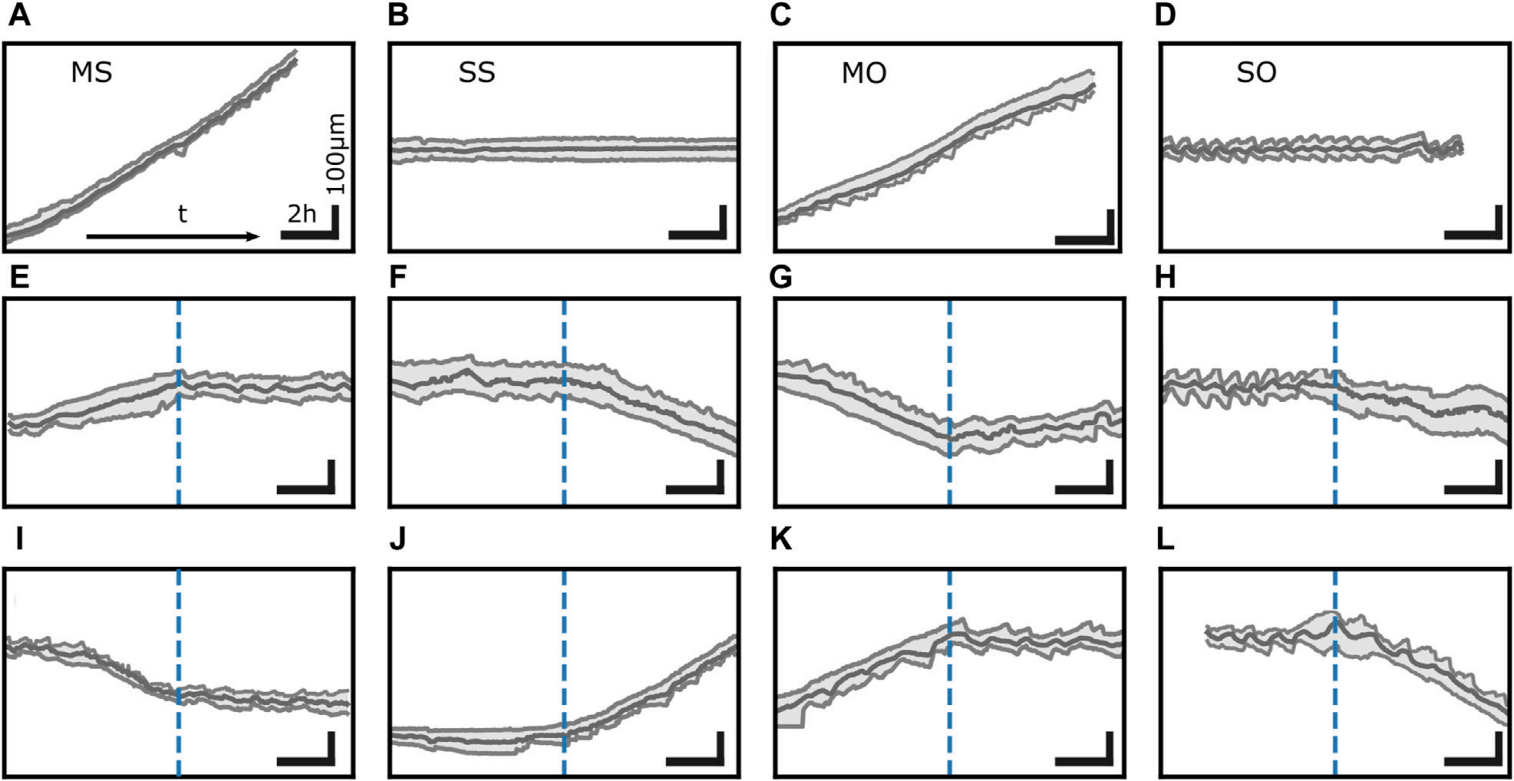
Transitions between distinct states in trajectories. MDA-MB-231 cells exhibit 4 dynamic states: **(A)** moving state with steady length (MS), **(B)** spread state with steady length (SS), **(C)** moving state with an oscillating back protrusion (MO), and **(D)** spread state with oscillatory protrusions at both ends (SO). **(E–L)** is a collection of spontaneous transitions between these states. Time runs from left to right. Vertical dashed blue lines mark the point in time of the transition. Horizontal scale bar 2 h, vertical scale bar 100 µm. All trajectories depicted here were measured on single MDA-MB-231 cells on fibronectin lanes.

The interaction of the functional units described so far causes not only motion but also morphodynamics. Cells exhibit distinct states of migratory behavior even within the same cell population and same condition, see Figure 3. As mentioned above, cells on 1D adhesive lanes often have protrusions at both ends as motion of the back edge in the direction opposite to the front edge direction shows (Guetta-Terrier et al., 2015; Monzo et al., 2016; Hennig et al., 2020; Amiri et al., 2023). Trajectories of the positions of the nucleus and the front and back edge of the cell provide the velocity v and the cell length L. We can break trajectories into episodes of consistent v- and L-dynamics and group these episodes into four states. In one of them, the back edge oscillates while the front moves almost steadily (Guetta-Terrier et al., 2015; Monzo et al., 2016; Hennig et al., 2020; Ron et al., 2020; Amiri et al., 2023). Beside this moving oscillatory state (MO), a spread oscillatory state (SO) with both ends oscillating, a spread state with steady length (SS) and a moving state with steady length (MS) have been observed (Guetta-Terrier et al., 2015; Monzo et al., 2016; Hennig et al., 2020; Ron et al., 2020; Amiri et al., 2023). Spontaneous transitions between all states (Amiri et al., 2023) suggest that they coexist, i.e., cells on 1D lanes exhibit multistability of dynamic states. The transitions observed by Amiri et al. suggest coexistence of all possible pairs of dynamic states. Spontaneous direction reversals demonstrate that moving states in one direction coexist with the same dynamic state moving in the opposite direction.

Cell motility and morphodynamics exhibit at least two different time scales. The dynamics of individual protrusions happens on a time scale of a few tens of seconds to minutes with small amplitude events being typically faster than large amplitude protrusion and retraction cycles (Giannone et al., 2004; Machacek and Danuser, 2006; Gholami et al., 2008; Koestler et al., 2008; Machacek et al., 2009; Enculescu et al., 2010; Burnette et al., 2011; Ron et al., 2020; Amiri et al., 2023). The interplay of polymerization, retrograde flow, F-actin network structure dynamics and membrane tension as well as local signaling have been related to this time scale. Cells show state transitions on a longer time scale. Restricting motion to 1D makes this very obvious since dynamic states can be more easily identified (see below) and direction changes occur between the discrete states “moving left” and “moving right”. The trajectories in Figures 1, 3 show both time scales. The oscillatory states SO and MO illustrate the shorter time scale of the dynamics of individual protrusions. The state transitions between dynamic states and direction reversals illustrate the slower state dynamics.

Confinement to 1D forces motile cells to revisit previously covered paths. This supports re-modelling of the micropatterned surface due to protein secretion, specifically fibronectin. Recently, it has been shown that a biochemical footprint is deposited as a consequence of secretion activity of migrating cells. It results in a memory effect which biases migration in a time-dependent manner (d’Alessandro et al., 2021; Perez Ipiña et al., 2023). Motion of cells shuttling forth and back between the ends of the high fibronectin region has been reported by d’Alessandro et al. (2021) as a consequence of secretion. However, it must be noted that these memory effects predominantly occur if cell lines are used that strongly secret and if initial protein concentration on the 1D lanes is low. In case of the tracks shown in Figure 3 for MDA-MB-231 cells on lanes within a range of 50–100 ng cm^-2^ fibronectin, there is no memory effect observable. A very similar effect can be obtained with non-secreting cells by the lane length. Cells turn around when they reach the end of short lanes (Zhou et al., 2020). The time scale of polarization is shortened when cells migrate into non-adhesive ends. The quenching and repolarization in the opposite direction results in a quasi-periodic motion with periods determined by lane-length and time of reversal.

## 5 What can biophysical modeling contribute to understanding motility on 1D lanes?

Modeling of cell mechanics and motility is a well-developed field. The specific mathematical model of individual studies is determined by the aspects of cell behavior and cell properties under consideration and the biological hypotheses on motility and morphodynamic mechanisms formulated by the model. Models focusing on the statistics generated by the trajectory of cell motion formulate the motion as persistent random walk (PRW) (Selmeczi et al., 2005; Romanczuk et al., 2012; Vestergaard et al., 2015; Shaebani et al., 2020; Zöttl and Stark, 2023) and may also take memory in the velocity dynamics into account (Mitterwallner et al., 2020). Other models formulate hypotheses on the intracellular mechanisms causing and controlling motility and morphodynamics. They usually take both signaling and mechanics into account but may appoint different roles to them. Some models hypothesize dynamics such as protrusion formation and shape oscillations to arise from signaling especially by small GTPases (Danuser et al., 2013; Rappel and Edelstein-Keshet, 2017; Bolado-Carrancio et al., 2020; Shaebani et al., 2020). Another group of models considers the signaling state of the cell as constant, setting the parameters for a dynamic mechanical system of cytoplasmic and F-actin flows, Myosin II based contraction and membrane dynamics (Kruse et al., 2005; Safran et al., 2005; Jülicher et al., 2007; Gholami et al., 2008; Enculescu et al., 2010; Zimmermann et al., 2010; Shaebani et al., 2020; Link et al., 2023). The modeling approaches have been described in several reviews (Romanczuk et al., 2012; Ryan et al., 2012; Danuser et al., 2013; Alert and Trepat, 2020; Shaebani et al., 2020; Link et al., 2023; Zöttl and Stark, 2023). Here, we focus on modeling studies specifically for 1D motion.

The dynamics on the long time scale has been addressed with a variety of modeling concepts. It determines the long term statistics like the UCSP and the dependency of the mean squared displacement (MSD) on time. Chemotaxis, durotaxis and the adhesion-velocity relation are properties of long-term velocity averages and thus belong also to the long time scale. Other studies focus on the shorter time scale of protrusion dynamics. Finally, we consider studies which attempt to explain the emergence of the long term dynamics from the protrusion dynamics.

### 5.1 The long time scale of state transitions

**FIGURE 4:**
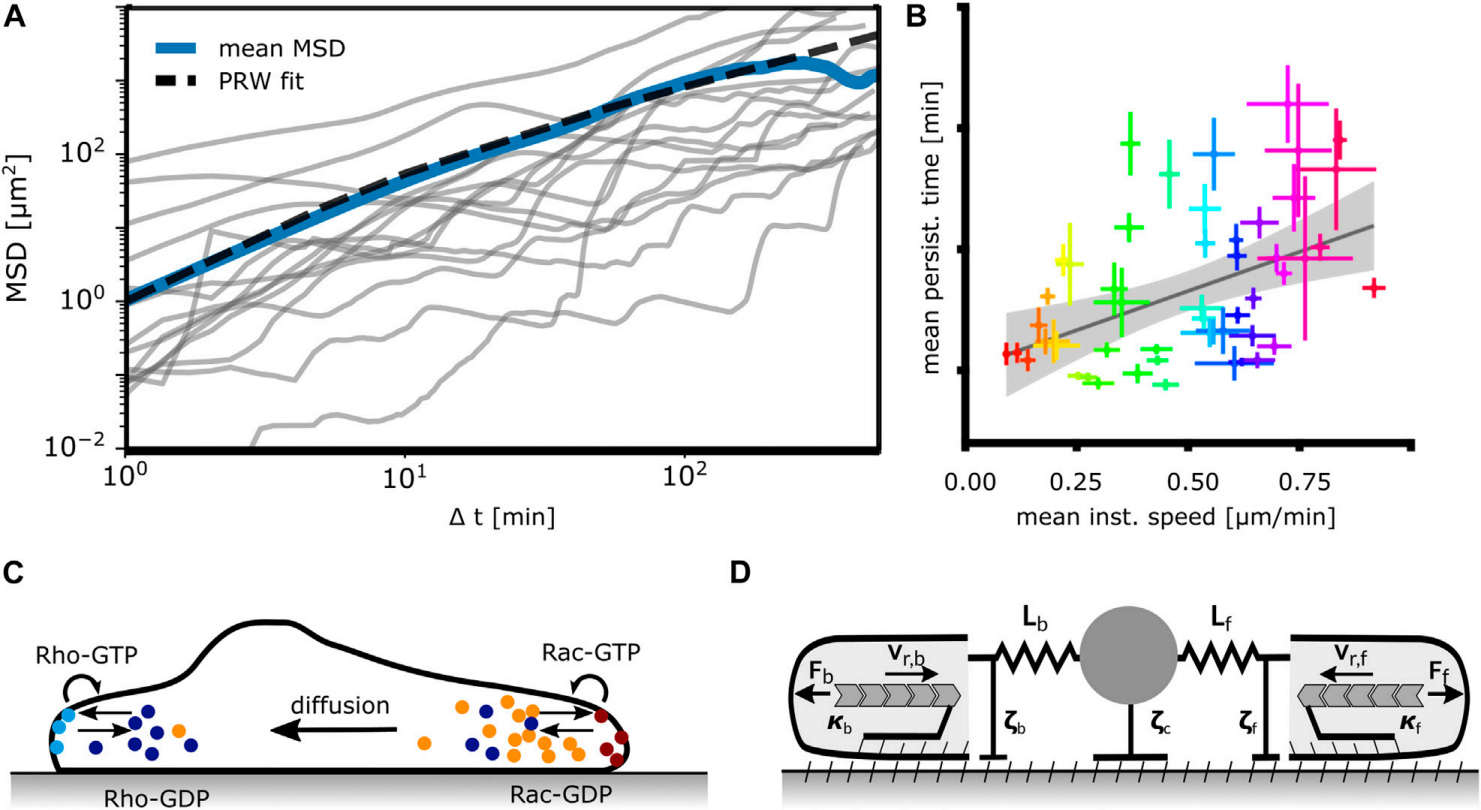
Mathematical models of mesenchymal cell migration. **(A)** Models perceiving cells as active Brownian particles moving randomly analyze the mean squared displacement (MSD) of trajectories such as the ones depicted here from MDA-MB-231 human breast cancer cells, N = 15. The blue line represents the mean MSD of the population, the dashed line represents a fitted persistent random walk model (PRW) with persistence time t_p_ = 5 min. **(B)** Universal coupling between cell speed and persistence (UCSP). Each dot indicates the mean persistence time and mean instantaneous speed of 1 cell line. Adapted from Maiuri et al. (2015). **(C)** Mathematical models based on signaling networks rely on a description of chemical reactions, transport and diffusion of reactants and consider concentration profiles of pathway components. Shown here is a prototypic system of bound/unbound Rho-GTPases. **(D)** Mechanical models formulate cellular dynamics in terms of forces (F_f,b_), drag (ζ_f,c,b_) and friction (κ_f,b_), F-actin network flow (v_r,f,b_) and membrane tension (elastic springs) acting on protrusions and the cell body. Adapted from Amiri et al. (2023).

We consider first models perceiving cells as active Brownian particles, i.e., as moving randomly with an intrinsic velocity, see Figure 4A. Most of the studies on cells as active Brownian particles focus on MSD scaling with time and velocity distributions.

Mitterwallner et al. (2020) investigate the validity of the concept of persistent random walk in cell motility. To this end, the velocity dynamics is described by a Langevin equation with an arbitrary memory kernel. It turns out that the memory of cells moving on circular micro-lanes exhibits a small negative friction indicating velocity control mechanisms at short time scales. Beyond that time, a persistent random walk describes cell behavior. The persistence time of individual cells derived from measured trajectories scatter over more than two orders of magnitude, which illustrates a large behavioral variability between cells.

Persistent random motion has also been observed with U251 glioblastoma cells moving on nano-fibers with 400 nm diameter (Estabridis et al., 2018). Cells moving in 1D were faster than on 2D fiber networks. Interestingly, U251 cells on 2D substrates did not exhibit persistence of their random motion. Chan and Odde developed a motor clutch model in the context of modeling filopodia behavior and applied it to cell motion on nano-fibers (Chan and Odde, 2008; Estabridis et al., 2018). The model explains the persistent random walk characteristics (Estabridis et al., 2018), and that motion on 2 parallel fibers is more persistent than on 1 fiber due to an increase in adhesion sites. It also exhibits the loss of persistence and smaller velocity in 2D due to adhesion strength in a range hindering motion, and offers an explanation for durotaxis (Bangasser et al., 2013) and the adhesion-velocity relation of glioma cells on 1D fibers (Klank et al., 2017). Clutches are modeled as individual spring-like bonds between F-actin and substrate. Retrograde flow results from the action of myosin motors on F-actin in this model. Retrograde flow and cell velocity are determined by the force-velocity relation of the myosin motors. Retrograde flow stretches the clutches and displaces clutch attachment points on the elastic substrate. Clutch dissociation rate from F-actin increases exponentially with the forces stretching the clutch. The sum of all clutch forces pulls the cell body which resists motion because it is also connected to the substrate by its own clutches (Chan and Odde, 2008; Bangasser et al., 2013; Estabridis et al., 2018). The sum of clutch length and substrate strain is equal to the oscillation amplitude in the oscillatory regime of this motor clutch model. All individual clutches disengage synchronously once a critical force is reached (Bangasser et al., 2013).

A modification of the PRW model has been used by d’Alessandro et al. to describe their observation that cells like to stay on the fibronectin layer which they deposited during their motion d’Alessandro et al. (2021). This has been modeled by increasing the probability to enter regions which the cell has visited earlier, and where it deposited fibronectin. This model is a form of a persistent self-attracting random walk (PSATW). If the cell reaches the boundary of the domain visited before, it is more likely to turn around at the boundary than crossing it. This leads to motion commuting forth and back between the ends of that domain. The domain grows with each visit of a cell at a boundary in experiments. In the simulations of the PSATW, the domain grows when the cell crosses its boundaries. The PSATW concept could reproduce the experimentally observed trajectories.

The constitutive relations as phenomena on the long time scale have been addressed by several studies. Maiuri et al. suggested that the UCSP exhibits an exponential dependency of the persistence time on the cell velocity, see Figure 4B (Maiuri et al., 2015). The purpose of the modeling part of the study was to support this hypothesis. It suggests intracellular processes causing the UCSP, and starts from two crucial assumptions (Maiuri et al., 2015). The first one assumes that the polymerization rate, which determines the retrograde flow velocity in the cell frame of reference, is controlled by a polymerization inhibitor binding to F-actin. Due to this binding, it is advected away from the leading edge membrane by retrograde flow. If this inhibitor reaches a critical value due to random behavior, leading edge motion stops. The statistics of this stochastic process then realizes the UCSP. The F-actin binding polymerization inhibitor has not been specified. Maiuri et al. derive an exponential relation between persistence time and retrograde flow velocity based on this assumption of an advected inhibitor. The second crucial assumption of this model is that the cell velocity v is proportional to the retrograde flow velocity v_r_ like v = αv_r_ with α being constant in the whole range of velocities to which the UCSP applies. In that case, the persistence time depends exponentially on cell velocity, too.

In our study by Schreiber et al. we investigated the adhesion-velocity relation and adhesion-cell length relation for MDA-MB-231 cells on fibronectin lanes (Schreiber et al., 2021). The modeling part derives an analytic expression for the adhesion-velocity relation from the force balance and the force dependency of the polymerization rate. Since only steady motion and long-term averages, respectively, are considered, linear friction for retrograde flow was sufficient for this study. Integrin signaling enters this model by functions describing the response of drag and friction coefficients to the fibronectin density. The adhesion-velocity relation measured for the MDA-MB-231 cells and relations for other cells can all be very well fit by the suggested equation for the adhesion-velocity relation. As a rule of thumb, the larger the ratio of the friction coefficient to the drag coefficient the larger is the cell velocity (Schreiber et al., 2021). The change of this ratio with changing fibronectin density dominates the adhesion-velocity relation (Schreiber et al., 2021). It increases in its rising phase. Signaling to the friction coefficient saturates slightly beyond the maximum velocity. The drag coefficient increases faster than the friction coefficient and their ratio decreases, which causes the falling phase of the adhesion-velocity relation and its saturation (Schreiber et al., 2021).

Individual cell types differ in the response of drag and friction coefficients of F-actin and membrane to integrin signaling. CHO cells are the slowest moving cells and exhibit the largest drag and friction coefficients, Keratocytes are the fastest cells and exhibit the smallest coefficient values (Schreiber et al., 2021). Fits of the adhesion-velocity relation to experimental data revealed that application of Blebbistatin substantially decreased the effect of fibronectin on the friction and drag coefficients. That may indicate that feedback by contraction is crucial for the formation of adhesion structures as suggested earlier (Gupton and Waterman-Storer, 2006; Even-Ram et al., 2007; Pasapera et al., 2010). An important conclusion from this study is that all forces affecting the cell velocity depend on the cell velocity and vanish when the velocity is 0. This explains how cells can move from high adhesion areas to low adhesion ones, since a force resisting motion in that direction occurs only when the cell is already moving.

Some studies perceive the dynamics on the long time scale, i.e., stretches of trajectories like the second half of the kymograph in Figure 1 with its 5 direction reversals, as deterministic oscillations. That has consequences for the model design. If the dynamics on this time scale is considered as random, the noise inherent to the processes on the short time scale determines the probability for direction reversal events (causing the UCSP) or state transitions. Thus, the noise determines the long time scale. If they are considered as deterministic, a process setting the long time scale is required.

Camley et al. (2013) motivate their study mainly with the observation of periodic motion of zyxin-depleted HT-1080 fibrosarcoma cells by Fraley et al. (2012) on 1D fibers in 3D networks and 1D stripes. Lavi et al. (2016) re-analysed trajectories of dendritic cells (DCs) in microchannels recorded by Chabaud et al. (2015) as periodic and refer to periodic motion of DCs reported by Solanes et al. (2015).

Camley et al. suggest the interaction between cell length dynamics driven by myosin-based contraction with a diffusing actin polymerization promoter to be the process setting the long time scale (Camley et al., 2013). Small GTPases define a reaction-diffusion system which can polarize the cell with high polymerization promoting concentration of active Rho protein at the front membrane of the cell and very low concentration at the back (Mori et al., 2008; Holmes et al., 2017; Rappel and Edelstein-Keshet, 2017). This mode of polarization requires a minimal cell length (Mori et al., 2008). The essence of the oscillations in the models by Camley et al. is the sequence of the cell length falling below this minimal length due to contraction, depolarization of the cell, relaxation of the length, repolarization and again contraction shortening the cell (Camley et al., 2013). This sequence sets the long time scale.

Dendritic cells accumulate vesicles at the cell front and these vesicles recruit myosin to the front (Chabaud et al., 2015; Lavi et al., 2016), i.e., there is a process counteracting the formation of the myosin gradient by advection by F-actin retrograde flow. Lavi et al. show that the complex interaction of the spatio-temporal myosin and vesicle dynamics with the action of myosin on F-actin flow can explain the periodic and stop-and-go motion of DCs in microchannels.

### 5.2 The short time scale of protrusion dynamics

Bolado-Carrancio et al. (2020) investigated the dynamics of a signaling network comprising the small GTPases Rac1 and RhoA, the kinases ROCK and PAK, and Diaphanous related formin-1 (DIA). This network exhibits oscillations in the concentration of GTPases in different states and thus also in the activation of polymerization and contraction. The complex feedbacks of the network allow for different state cycles. A RhoA/Rac1 cycle dominates the signaling at the cell front, a RhoA/ROCK cycle the signaling at the back of a model cell (Figure 4C) (Bolado-Carrancio et al., 2020). That entails protrusion retraction cycles at the front edge with a period of about 1 min and adhesion-retraction cycles at the rear with a period of 5–10 min. These observations are different from the oscillations of MDA-MB-231 cells observed in Amiri et al. (2023). Protrusion activity is symmetric in the spread state and oscillations occur at the back only when the cell is moving, and periods are in the range of 10 min–60 min (Amiri et al., 2023).

Simplified mechanistic descriptions of cell mechanics formulate the dynamics starting from force balance, low Reynolds number flow equations and the constitutive equations of the F-actin network and F-actin polymerization, see Figure 4D. The constitutive equation for friction between the retrogradely flowing F-actin network and structures stationary in the lab frame of reference is the non-linear friction of the clutch. Chan and Odde modeled it in a discrete way as explained above (Chan and Odde, 2008; Bangasser et al., 2013; Estabridis et al., 2018). The bowed instruments example of stick-slip dynamics illustrates that there is also a continuous regime in which the strain on bonds is much smaller than the oscillation amplitude and slippage occurs due to the increase in dissociation rate. Stick slip models formulated in terms of continuous bond fractions work in that regime, e.g., (Gerbal et al., 2000; Bernheim-Groswasser et al., 2005; Craig et al., 2015; Hennig et al., 2020). Three studies Ron et al. (2020); Sens (2020), and our work (Amiri et al., 2023) include the clutch mechanism as an oscillation mechanism and report on emerging multistability of dynamic states.

The theoretical study by Sens puts forward a model that comprises two protrusions linked by an elastically behaving membrane (Sens, 2020). The model by Ron et al. is based on Maiuri et al. and assumes a polymerization inhibitor diffusing in the cytosol and binding to F-actin controlling polymerization in two protrusions (Ron et al., 2020). Advective transport of this inhibitor by retrograde flow away from the edge membrane stabilizes the protrusion. The concentration profile of this inhibitor polarizes the cell. In difference to that, polarization arises from protrusion competition mediated by membrane tension and the clutch mechanism in the studies by Sens and Amiri et al., resp. (Sens, 2020; Amiri et al., 2023) very similar to the results by Hennig et al. (2020). Ron et al. provide qualitative comparisons of dynamic cell states with data from C6 glioma cells and fibroblasts. In our work, we generalized the model used in Schreiber et al. to include the clutch of retrograde flow friction, the cell body, and protrusions at both cell ends (Schreiber et al., 2021; Amiri et al., 2023). We parameterized the model by experimental data and provided detailed quantitative comparisons between experiment and theory including state transitions.

All three models exhibit the states spread steady SS, moving oscillatory MO and moving steady MS. The models by Amiri et al. and Sens show the noise free state MO with in phase oscillations of both protrusions, the model by Ron et al. with antiphase oscillations. MDA-MB-231 cells exhibit noisy oscillations and sometimes oscillations only slightly perturbed by noise, which are in phase (Amiri et al., 2023). Noisy oscillations do not exhibit a specific phase relation (Amiri et al., 2023). Noisy oscillations occur in the oscillatory regime and an excitable regime of the states MS and SS (Amiri et al., 2023). All three models exhibit coexistence of at least some of the possible dynamic state pairings (Ron et al., 2020; Sens, 2020; Amiri et al., 2023). Hence, the dynamic states and multistability appear to be intrinsic to cell models comprising two (or more) protrusions with the clutch mechanism and coupled by membrane tension.

### 5.3 Relating short and long time scale

Both the mean squared displacement behavior and the UCSP as properties on the long time scale are statistics of random motion. That suggests them to be generated by the randomness on the short time scale. Estabridis et al. had shown that motion generated by their motor clutch model (short time scale) reproduces the characteristics of a persistent random walk (long time scale) (Estabridis et al., 2018). Is there a mechanism explaining additionally the dynamic cell states and the UCSP?

**FIGURE 5:**
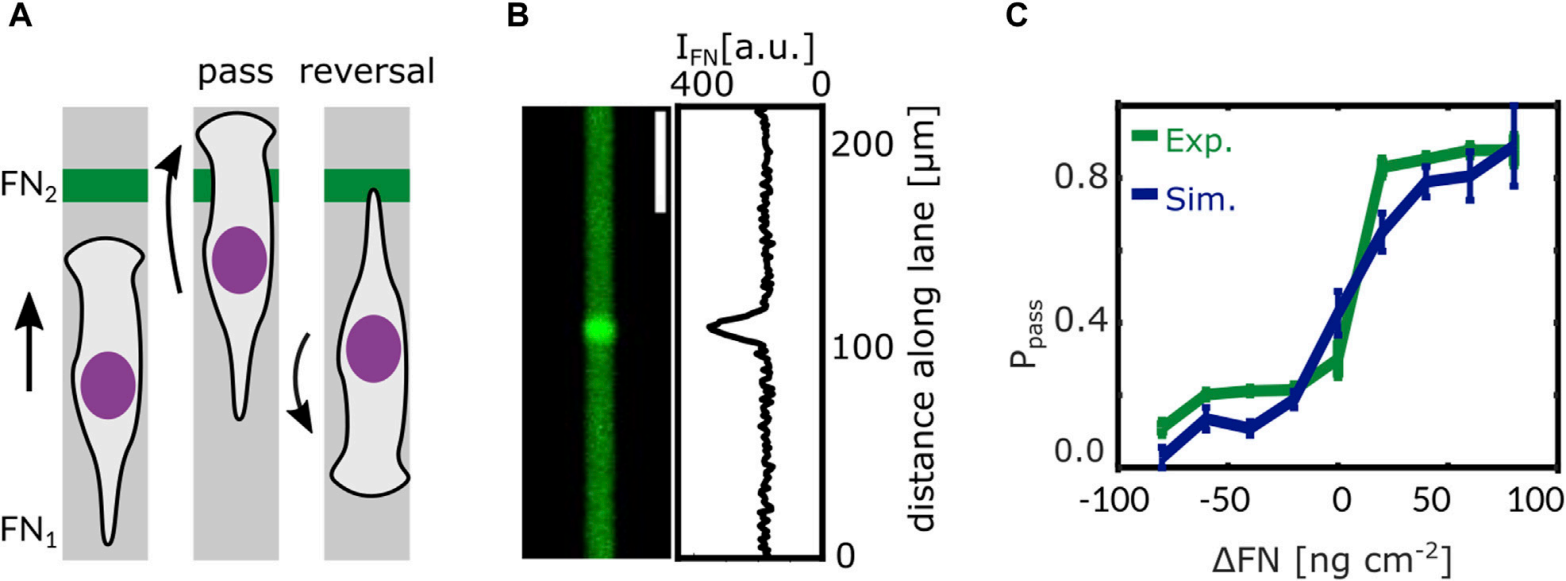
Steps in ligand density reveal the integrin signaling response function. **(A)** When a cell on a 1D microlane encounters a stepwise change in ligand density its response is one of the following scenarios: either the cell passes the step or it re-polarises and reverses direction. **(B)** Fluorescent image of a microlane of Alexa 647 labeled Fibronectin (FN) and corresponding gray value intensity profile along the lane. The intensity of the fluorescent signal is proportional to the local FN density. A stepwise change in FN density can be seen in the center of the lane. **(C)** Models predict that the probability P_pass_ of a cell to pass the step depends on the difference in fibronectin density ΔFN as has been confirmed by experiment. Adapted from Amiri et al. (2023).

In our study by Amiri et al. we suggest such a mechanism. The model formulating it is the same as for the short time scale introduced above. It has been developed on the basis of data on protrusion dynamics and of a large amount of MDA-MB-231 cell trajectories allowing for quantifying the statistics of state transitions on the long time scale (Amiri et al., 2023). The model used one set of parameters to describe all control experiments. The network extension rate has been reduced to describe all experiments with Latrunculin application. All Blebbistatin experiments were described by changes of the control parameter value set as suggested by fits of the adhesion-velocity relation by Schreiber et al. (2021). The integrin signaling response functions have been parameterized by reproducing cell behavior at fibronectin density steps in terms of the probability of a cell passing the step, see Figure 5 (Amiri et al., 2023).

State transitions are the experimental manifestation of multistability. Since they were observed without any intervention or obvious perturbation, they are assumed to be spontaneous and to be due to noise. In our model we assume the friction coefficient of retrograde flow to be the random variable due to noise from the formation and rupture of bonds between the retrogradely flowing F-actin network and structures stationary in the lab frame of reference. Adhesion sites are very complex structures. The model does not specify which of the several intermediate bonds linking F-actin to the substrate breaks (or forms). It might be any bond from fibronectin dissociating from the substrate to an F-actin binding component of an adhesion complex dissociating from F-actin. The state fraction statistics and the state transition statistics can both be used to verify assumptions on noise source and strength. We obtained good agreement between experiments and simulations for the state fraction statistics of control experiments and with Latrunculin or Blebbistatin applied (Amiri et al., 2023). State transition statistics provide the fractions of transitions out of a given state to each of the other states. This statistics is also satisfyingly reproduced for control experiments and both drugs applied by the assumptions of noise in the friction coefficient due to bond dynamics (Amiri et al., 2023).

The UCSP is the statistics of direction reversal events. Hence, it can serve to verify the direction reversal mechanism and the modeling choices for noise. The model with protrusion competition mediated by membrane tension and the clutch mechanism as direction reversal mechanism and noise in the friction coefficient of the clutch initiating direction reversals could reproduce the UCSP-relations of MDA-MB-231 cells in control experiments and with Latrunculin or Blebbistatin applied. Surprisingly, application of Latrunculin increased persistence of MDA-MB-231 cell motion. Since Latrunculin inhibits polymerization (Coué et al., 1987), inhibition of polymerization appears not to reduce persistence. These results appear to be difficult to reconcile with the advected inhibitor mechanism for which a reduction of the polymerization rate should destabilize protrusions and reduce persistence.

In summary, the clutch mechanism of retrograde flow, polymerization and membrane tension form a mechanical system exhibiting the multistability of dynamic cell states. Noise in the friction coefficient from random bond dynamics causes state transitions. Protrusion competition and friction noise cause direction reversals explaining the UCSP. Integrin signaling sets the parameters of this system and thus the dynamic regime, and explains the adhesion-velocity relation.

As final note, the response of the cell velocity to a constant force resisting motion is the stationary force-velocity relation and most likely a third constitutive relation of cell motility. While the response to an elastic force is not a constitutive relation but has been well investigated and understood (Prass et al., 2006; Heinemann et al., 2011; Zimmermann et al., 2012), the stationary force-velocity relation has not been measured, yet. Theoretical predictions suggest that it would reflect the velocity-friction force relation of retrograde flow (Zimmermann et al., 2010; Amiri et al., 2023). A linear friction law would entail a linear stationary force-velocity relation with negative slope intersecting the force-axis at the stall force; the clutch mechanism causes small deviations from the linear relation.

## 6 Conclusions and perspectives

We reviewed cell migration in 1D confinement showing that quantification of low-dimensional morphodynamics in conjunction with biophysical modeling provides unique opportunities to unravel mechanisms of mesenchymal migration. As envisioned in the seminal announcement of the “cell race” (Maiuri et al., 2012), 1D microlanes provide a standardized platform enabling high-throughput acquisition of 1D cell trajectories. Stratified experiments mapped the behavior of various cell types measured in different labs and led to the discovery of universal properties as well as assessment of cell-type specific parameters. Although motion is restricted to 1D, the dynamics of front and rear exhibit a surprising complexity. Cell trajectories display characteristic states of motion and different cell types exhibit different 1D migratory phenotypes. Much of the observed cell dynamics is reproduced by recent mechanical models that implement the force balance within the force generating mechanisms in the cytoskeleton including actin polymerisation, retrograde flow, membrane tension, integrin mediated molecular clutch and signaling. The models lead into concepts of non-linear dynamics, in particular the concept of multistability of dynamic systems. The latter explains the variety of observed dynamic states and state transitions, and the constitutive relations.

The full spatio-temporal distribution of cytoskeleton activity along the 1D contour will be measured in future experiments. Quantities of interest are the focal adhesion density, actin state and distribution as well as concentrations of regulator proteins. In this context also the advent of traction force microscopy is seminal as it technically enables measurement of spatial 1D force profiles in 1D microlanes (Gardel et al., 2010; Vignaud et al., 2012; Schwarz and Soiné, 2015).

Large data sets of trajectories and density profiles are a powerful basis for statistical analysis. Unsupervised learning will be used to generate classifiers in cell migration time series in future AI driven analysis. Neural networks can be trained by simulated data and used to detect migration states in order to compare data and computational models. The demand for even larger data sets of cell behavior will grow as neural network based analysis and cell migration models advance. One possible approach to generate more information from motile cells are multiple experiments under standardized conditions with defined perturbations. The challenge then is to describe morphodynamics under a series of conditions in a self-consistent manner.

**FIGURE 6:**
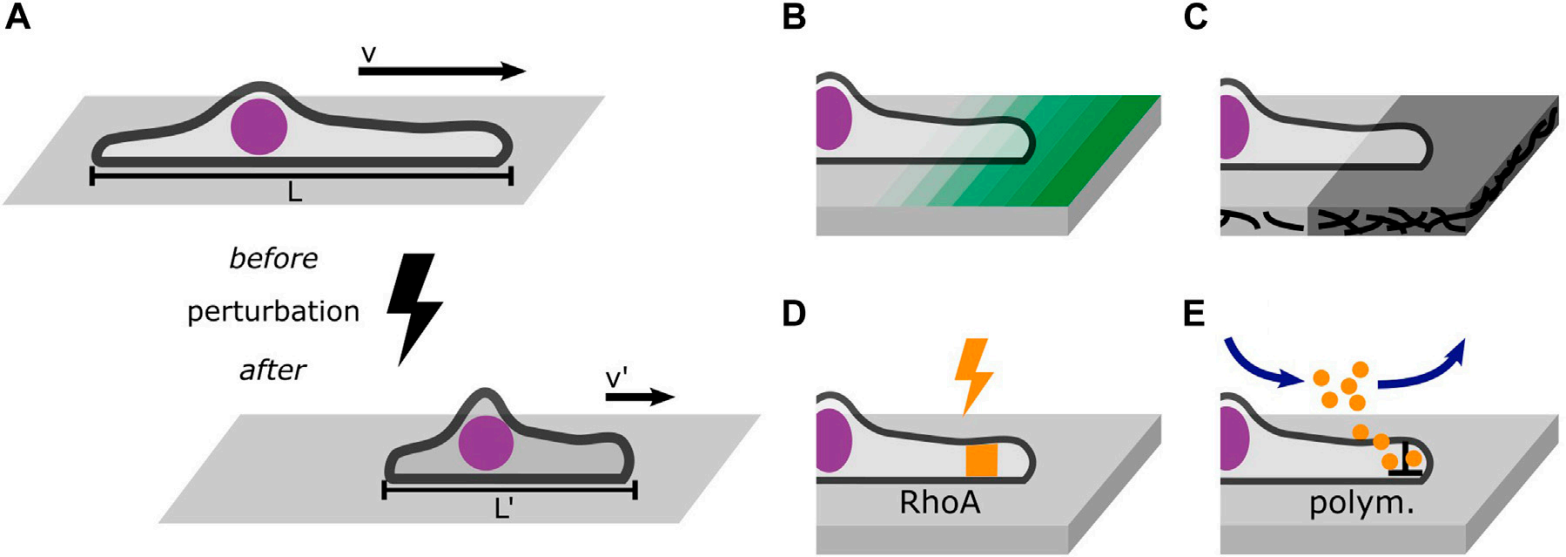
Perturbations probe the cell’s response to changes in its environment. **(A)** Well-defined perturbations can be placed in a cell’s path to probe its response such as migration velocity or morphology. **(B)** Gradients of ligand density probe the cell’s response to changing adhesion strength. **(C)** Steps in the stiffness of the adhesive substrate reveal information about the role of trajection forces in mesenchymal migration. **(D)** Optogenetic tools can dynamically activate and inactivate the RhoA pathway which affects cell contractility. **(E)** Drug treatment mediated by microfluidics allows the continuous observation of cells before and after exposure. A worthwhile target of drug treatment is the polymerization of actin filaments which can be inhibited using Latrunculin **(A)**.

In addition, time-resolved perturbations in 1D microlane assays represent an experimental approach to probe the dynamic response of cells as depicted in Figure 6. In general, perturbation of migratory behavior has been employed in many studies on cell migration. Optogenetic studies probe the time-resolved response to changes in cell contractility induced by light-induced activation of the RhoA-pathway (Oakes et al., 2017; Valon et al., 2017; Hennig et al., 2020; Drozdowski et al., 2023). Optical manipulation using UV flood exposure also allows for the release of caged molecules (Ellis-Davies, 2007) or dynamic control of the micro-environment by removal of surface coating (Rolli et al., 2012; Vignaud et al., 2012). However, there are few studies where perturbations are evaluated at the single cell level comparing trajectories before and after the perturbation, Figure 6A. The study of single cells crossing adhesion steps on microlanes directly compares the impact of adhesion strength, see Figure 6B. In principle, also the effects of drugs could be studied in a time-resolved manner, if appropriate microfluidic flow chamber devices combined with time-lapse live cell imaging allow the continuous observation of cells migrating on 1D microlanes before and after exposure, see Figure 6E. Likewise ligand induced changes in cell state are detectable *in situ* (Copperman et al., 2023). The addition of cytoskeleton inhibitors represents the most established biochemical manipulation of cell migration. Combining inhibitors with 1D microlane studies could resolve single cell response to molecular targeting. Advancement in nano- and micro-structuring, including bioprinting or dip-pen-nanolithography (Salaita et al., 2007; Wu et al., 2011) offer yet unexplored possibilities to expose cells to molecular cues that impact migration. In particular, multi-protein printing and the coating of protein gradients (Ricoult et al., 2015) will enhance the assessment of migratory cell response. The combination of these methods with 1D microlane assays provides a well-defined encounter of cells with geometrically restricted perturbations. However, the defined deposition of ECM proteins in terms of protein density and protein conformation remains challenging. In particular large proteins like fibronectin tend to unfold depending on surface properties and protocols for deposition. Micropatterning of ECM protein on soft hydrogel substrates allows for traction force microscopy in 1D microlanes as demonstrated by Hennig et al. (2020). The collectivity of perturbation experiments and variations provide an amount of trajectories that contain sufficient statistics to systematically characterize single cell dynamic responses.

In summary, 1D migration assays are a productive tool to standardize the characterization of migratory phenotypes and the examination of theoretical models. Cell behavior in a simplified geometry is well quantifiable and allows for in depth comparison with theory. The dimensional reduction also provides the means for integration of multiple observables, including force fields or protein concentration profiles. In addition, time-dependent perturbations challenge theoretical models to predict dynamic response of migratory behavior in variable environmental conditions. In view of the rapid development of AI-based analysis, the establishment of migratory data repositories is desirable and will possibly pave the way for usage of AI-based 1D migratory assays in drug screening. As such the outcomes are likely to be relevant to more physiological conditions. Ultimately, massive 1D migration data will refine our understanding of mechanisms governing mesenchymal cell motion.
